# The Low Expression of Fc-Gamma Receptor III (CD16) and High Expression of Fc-Gamma Receptor I (CD64) on Neutrophil Granulocytes Mark Severe COVID-19 Pneumonia

**DOI:** 10.3390/diagnostics12082010

**Published:** 2022-08-19

**Authors:** Joerg Hoffmann, Rojin Etati, Cornelia Brendel, Andreas Neubauer, Elisabeth Mack

**Affiliations:** Department of Hematology, Oncology and Immunology, University Hospital Giessen and Marburg, Philipps University Marburg, Baldingerstrasse, D-35043 Marburg, Germany

**Keywords:** COVID-19, corona, pneumonia, flow cytometry, CD16, CD64, Fc-gamma receptor

## Abstract

Hyperinflammation through neutrophil granulocytes contributes to disease severity in COVID-19 pneumonia and promotes acute lung failure. Understanding the mechanisms of the dysregulations within the myeloid cell compartment may help to improve therapies for severe COVID-19 infection. Here, we investigated the immunopathological characteristics of circulating neutrophil granulocytes and monocytes in 16 patients with COVID-19 pneumonia by multiparameter flow cytometry in comparison to 9 patients with pulmonary infiltrates but without COVID-19. We correlated the immunophenotypes with the scores of the severity-of-disease classification system, APACHE-II. We found that the mean fluorescence intensity (MFI) of CD15, which is important for the transendothelial migration, was significantly reduced in the patients with COVID-19 (difference ± SD; 295.70 ± 117.50 MFI; *p* = 0.02). In addition, the granularity was significantly lower in the neutrophil granulocytes of patients with COVID-19 (difference ± SD; 1.11 ± 0.43 side-scatter ratio; *p* = 0.02). Moreover, the Fc-gamma receptor III (CD16) and Fc-gamma receptor I (CD64) on the neutrophil granulocytes were expressed discordantly with COVID-19 severity. CD16 correlated as inversely proportional (ρ = (−)0.72; 95% CI (−)0.92–(−)0.23; *p* = 0.01) and CD64 as proportional (ρ = 0.76; 95% CI 0.31–0.93; *p* = 0.01) with the APACHE-II scores of the patients. We conclude that the deviant expression of the Fc-gamma receptors might play role in a dysregulated antibody-mediated phagocytosis in severe cases of COVID-19 pneumonia.

## 1. Introduction

The severe acute respiratory syndrome coronavirus 2 (SARS-CoV-2), the cause of coronavirus disease 2019 (COVID-19), appeared in December 2019 in Wuhan, China [1]. The fight against the COVID-19 pandemic and its consequences on health care systems and societies are issues of worldwide importance. A total of 574 million confirmed COVID-19 cases and 6.3 million deaths had reported globally by the end of July 2022 [2]. In the U.S., between January and May 2020, about 14% of all COVID-19 cases required hospitalization, 2% were admitted to an intensive care unit (ICU), and 5% resulted in death [3]. The outbreak of the Omicron variant in November 2021 resulted in decreased death rates (4.5% vs. 21.3%) and ICU admissions (1% vs. 4.3%) compared to the previous waves in South Africa [4].

COVID-19 can result in acute respiratory distress syndrome (ARDS) with rapidly progressive lung failure [5,6]. ARDS is a complication of the COVID-19 infection, with high morbidity and mortality. In particular, patients of older ages or with comorbidities are at an increased risk, and more often experience a difficult course, of disease [7,8,9,10]. A dysregulated immune response with cytokine-induced hyperinflammation contributes to the morbidity and mortality of COVID-19 [11,12,13]. Several studies have demonstrated favorable outcomes with the use of immune response modifiers in patients with severe COVID-19 infections [14,15,16,17,18,19].

A high neutrophils/lymphocyte ratio helps to predict severe COVID-19 [14,20,21]. After neutrophil granulocytes, monocytes are the second most abundant group of myeloid cells in the peripheral blood. Monocytes can be separated into at least three different subsets: the CD16 (Fc-gamma receptor III)-negative classical subset and the intermediate and non-classical CD16-positive subsets [22,23,24,25]. The CD16-positive subsets are more involved in antigen presentation and Fc-gamma-mediated phagocytosis than the classical monocytes [26,27]. In a comprehensive analysis, Schulte-Schrepping et al. described abundant alterations in the myeloid cell compartment associated with COVID-19, such as the predominance of inflammatory monocytes in mild cases of the disease, whereas dysfunctional monocytes occurred primarily in severe COVID-19 [28]. Important questions concerning the role of neutrophil granulocytes and monocytes in hyperinflammation and their therapeutic implications in COVID-19 remain to be elucidated.

The main objective of this study was to identify characteristics in the immunophenotype of the neutrophil granulocytes and monocytes that are distinctive of a COVID-19 infection and indicate a severe course of the disease. This discovery could aid in the clinical assessment of COVID-19 patients and improve our understanding of the role of immunopathological dysregulation in COVID-19. Therefore, we investigated the expression of cell surface proteins with key functions (i.e., adhesion, migration, extravasation, and phagocytosis) in the immune response of the neutrophil granulocytes and monocytes in patients with COVID-19 pneumonia and compared the findings to patients with pulmonary infiltrates but without COVID-19.

## 2. Materials and Methods

### 2.1. Patients

This study was performed at the beginning of the first wave of the COVID-19 pandemic in Germany, between March and May 2020, at a single academic center (University Hospital Marburg). A total of 30 patients with clinical symptoms of respiratory infection compatible with COVID-19 were enrolled. Peripheral blood samples were collected after written informed consent was obtained. The study was conducted according to the guidelines of the local ethics committee (Vote 57/20) and the Declaration of Helsinki. Pulmonary infiltrates identified on the CT scan were mandatory for inclusion in the final analysis. Patients were tested for SARS-CoV-2 infection with combined E- and S-specific PCR (RealStar^®^ SARS-CoV-2 RT-PCR Kit, Altona Diagnostics, Hamburg, Germany) using a nasopharyngeal swab and separated into a COVID-19-positive group and a control group.

We measured the expression of the cell surface proteins with respect to their adhesion, migration, extravasation (CD15, CD38, and CD11b), (antibody-mediated) phagocytosis and apoptosis (CD33, CD36, CD16, CD64, and CD13), and antigen-presentation (HLA-DR) via multiparameter flow cytometry (MPFC) applied to the neutrophil granulocytes and monocytes. In addition, the release of soluble anti-microbial granules (i.e., granularity) was quantified for the neutrophil granulocytes. The monocytes were analyzed for the expression of the pattern recognition receptor CD14, aberrant expression of the cell-to-cell adhesion molecule CD56, and the count of the CD16-positive monocyte subset (non-classical and intermediate). Immunophenotypic characteristics of the granulocytes and monocytes of the patients in intensive care units (ICU) with COVID-19 were aligned with the APACHE-II scores, which are prognostic for morbidity and mortality [29]. The severity of ARDS was assessed using the Horowitz index [30,31]. Other clinical parameters, such as age, number of days in the hospital or ICU, comorbidities (Charlson comorbidity index, CCI), inflammation (C-reactive protein and procalcitonin), and coagulation (d-dimer, thrombocytes, fibrinogen, prothrombin time, activated partial thromboplastin time, and thrombin clotting time) were correlated in relation to the immunophenotypic characteristics.

Sample handling, flow cytometry data acquisition, and analysis.

A total of 2 × 100 µL peripheral blood was transferred into two separate 5 mL polystyrene tubes with fluorescence antibodies in a dried-down layer (DURAClone Technology, Beckman Coulter, Brea, CA, USA) and incubated for 15 min at room temperature. The following antibody combinations were used for the immunological studies:

Tube 1: CD15 FITC (clone 80H5), CD33 PE (clone D3HL60.251), CD13 ECD (clone SJ1D1), CD117 PC 5.5 (clone 104D2D1), CD34 PC7 (clone 581), CD10 APC (clone ALB1), CD16 APC-AF700 (clone 3G8), CD11b APC-AF750 (clone BEAR1), HLA-DR Pacific blue (clone IMMU-357), and CD45 Krome orange (clone J33).

Tube 2: CD7 FITC (clone 8H8.1), CD19 PE (clone J3.119), CD38 ECD (clone LS198-4-3), CD56 PC5.5 (clone N901 (NKH-1)), CD34 PC7 (clone 581), CD10 APC (clone ALB1), CD14 APC-AF700 (clone RMO52), CD64 APC-AF750 (clone 22), CD36 Pacific blue (clone FA6.152), and CD45 Krome orange (clone J33) (all from Beckman Coulter).

After antibody staining, red cells were lysed in 2 mL Versalyse (Beckman Coulter) for 10 min, washed with 3 mL phosphate-buffered saline (PBS Biochrom, Berlin, Germany), and centrifuged with 300× *g* for 5 min. The cell pellet was resuspended in 500 μL PBS and measured on a Navios flow cytometer (Beckman Coulter). In total, up to 1 × 105 cells were acquired. The flow cytometry analysis and gating were performed with Kaluza vs. 2.1. (Beckman Coulter). Debris, red cells, thrombocyte aggregates were excluded via side-scatter (SSC), forward-scatter (FSC), and the pan-leucocyte antigen CD45. The lymphocytes, monocytes, and granulocytes were pre-gated via the two-dimensional SSC/CD45 plot. Mature neutrophil granulocytes were selected in the CD16/CD13 plot to exclude the eosinophils (CD16-negative) and immature granulocytes/myelocytes (CD13 dim). The monocytes were selected in the CD36/CD14 plot. The relative mean fluorescence intensity was determined using the ratio of the geometric mean fluorescence of the neutrophil granulocytes or monocytes and the geometric mean fluorescence of the negative population of the lymphocytes. The granularity was measured in each sample using the ratio of SSC neutrophil granulocytes/SSC lymphocytes (SSC ratio).

### 2.2. Statistics

The charts and statistics were compiled on Excel 2016 (Microsoft Corporation, Redmond, WA, USA) and GraphPad Prism^®^ Version 5.00 (GraphPad Software, San Diego, CA, USA). The relative MFI from CD15, CD38, CD11b, CD33, CD36, CD16, CD64, CD13, HLA-DR, CD14, and CD56, and granularity and percentages/counts for CD16-positive monocytes, were compared between patients with COVID-19 and without COVID-19 using the Mann–Whitney U test. Significantly different antigens (*p* < 0.05) were analyzed based on receiver operating characteristic (ROC) curves for the binary classification. Immunophenotypic characteristics were correlated with the clinical parameters using Spearman’s rank correlation coefficient. Results of the correlation coefficient ρ were depicted in a correlation matrix (heat map).

## 3. Results

### 3.1. Study Population and Patient Characteristics

**Figure 1:**
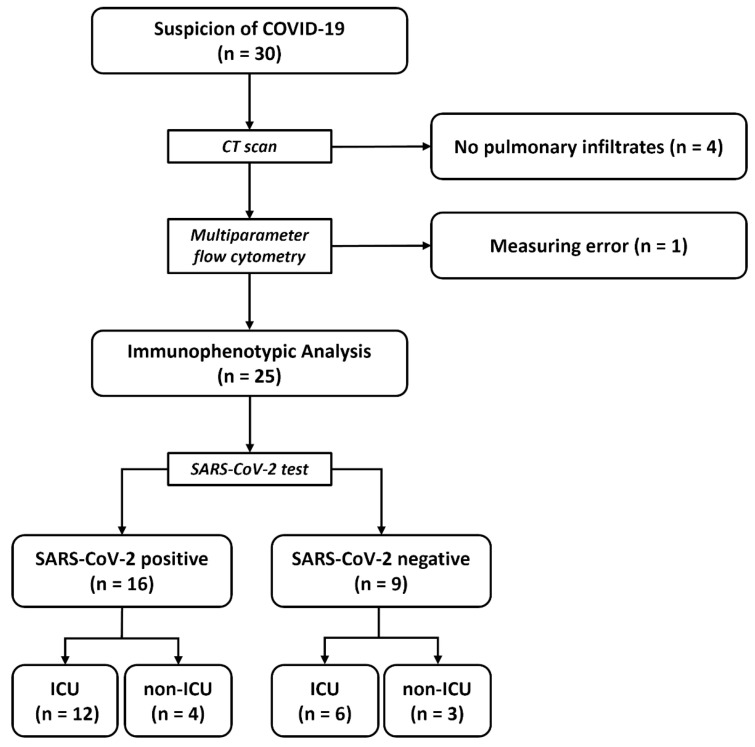
Study population and workflow. Abbreviations: *n* = number; ICU = intensive care unit, SARS-CoV-2 = severe acute respiratory syndrome coronavirus 2.

In total, 30 patients were hospitalized with suspected COVID-19 and enrolled in the study. CT scans revealed pulmonary infiltrates in 26 patients. The MPFC was performed without measurement errors in 25 patients, who were included in the final analysis of the immunophenotypes of myeloid cells (Figure 1).

The median age of the patients was 67 years (range 23–88 years) and 19% were female. The median duration of hospitalization was 28 days (range 7–67 days) and patients in the ICU received intensive care for a median of 19 days (range 2–49 days). A total of 21/25 (84%) patients were discharged from hospital and 4/25 (16%) patients died during hospitalization. At baseline, 16/25 (64%) patients had a positive SARS-CoV-2 PCR result and 18/25 (72%) were admitted to the ICU. In the ICU subgroup, 12 patients were positive for SARS-CoV-2 and 4 patients were negative, and in the non-ICU group, 4 patients were positive and 3 were negative for SARS-CoV-2. All nine patients who were negative for SARS-CoV-2 had an atypical form of pneumonia, but no causative pathogen could be found. Procalcitonin, as a marker of a bacterial infection, was elevated in four out of nine (44%) patients without SARS-CoV-2. Further patient characteristics are denoted in Table 1.

### 3.2. Immunological Differences between SARS-CoV-2-Positive and SARS-CoV-2-Negative Patients

**Figure 2:**
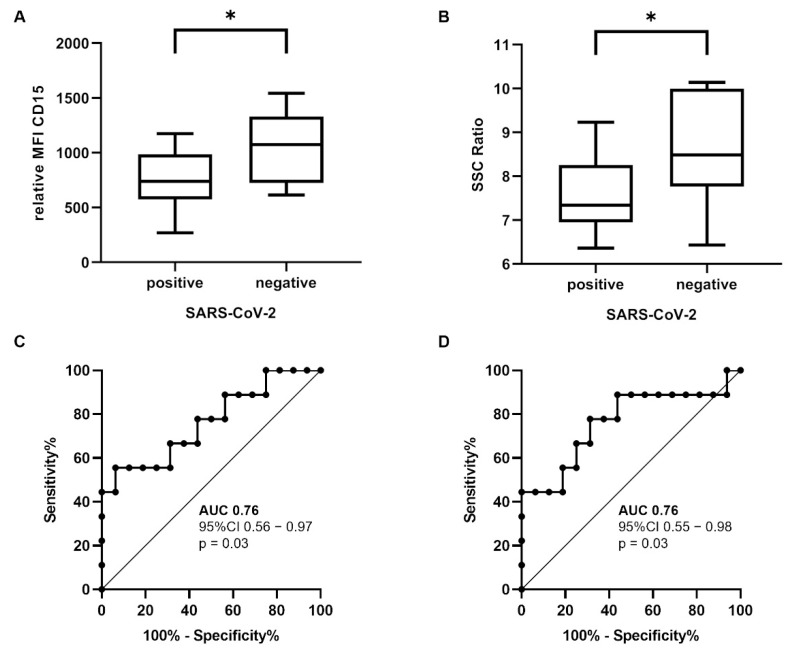
**Immunophenotypic differences of the circulating neutrophil granulocytes.** CD15 (**A**) and SSC ratio (**B**) were significantly reduced in patients with SARS-CoV-2 infection (difference ± SD; 295.70 ± 117.50 MFI; *p* = 0.02 and 1.11 ± 0.43 SSC ratio; *p* = 0.02). ROC curves showed an AUC of 0.76 for the CD15 (**C**) and SSC ratio (**D**). Mann–Whitney U test * *p* < 0.05 (abbreviations: *n* = number; MFI = mean fluorescence intensity; SSC = side-scatter; SARS-CoV-2 = severe acute respiratory syndrome coronavirus 2; AUC = area under curve; CI = confidence interval; ROC = receiver operating characteristic).

The neutrophil granulocytes in patients with a positive SARS-CoV-2 test (*n* = 16) had a significantly reduced CD15 expression (difference ± SD; 295.70 ± 117.50 MFI; *p* = 0.02) and reduced SSC ratio (i.e., granularity; difference ± SD; 1.11 ± 0.43 SSC-Ratio; *p* = 0.02) than patients with a negative SARS-CoV-2 test (*n* = 9) in the Mann–Whitney test (Figure 2A,B).

We performed an ROC analysis to examine whether CD15 and SSC are useful as binary classifiers for distinguishing between SARS-CoV-2-positive and SARS-CoV-2-negative patients (Figure 2C,D). CD15 had an area under curve (AUC) of 0.76 (95% CI 0.56–0.97; *p* = 0.03) and SSC had an AUC of 0.76 (95% CI 0.55–0.98, *p* = 0.03). This means that the CD15 and SSC of neutrophil granulocytes could discriminate between SARS-CoV-2-positive and SARS-CoV-2-negative patients in 76% of the cases.

**Figure 3:**
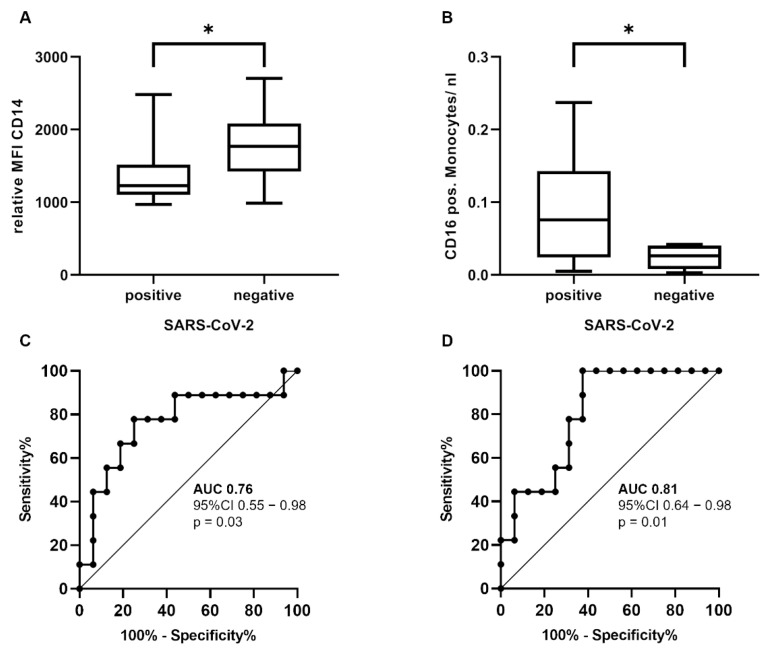
**Immunophenotypic differences of the circulating monocytes.** The CD14 expression (**A**) was significantly reduced (difference ± SD; 405.90 ± 177.40 MFI; *p* = 0.03) and the CD16 + monocyte count (**B**) significantly increased (difference ± SD; 0.07 ± 0.03 per nl; *p* = 0.01) in patients with SARS-CoV-2 infection. ROC curves showed an AUC of 0.76 for CD14 (**C**) and an AUC of 0.81 for the CD16 + monocyte count (**D**). Mann–Whitney U test with * *p* < 0.05 (abbreviations: *n* = number; MFI = mean fluorescence intensity; SSC = side-scatter; SARS-CoV-2 = severe acute respiratory syndrome coronavirus 2, AUC = area under curve; CI = confidence interval; ROC = receiver operating characteristic).

In the monocyte compartment, the expression of CD15 did not distinguish between SARS-CoV-2-positive and SARS-CoV-2-negative patients (*p* = 0.96). However, the CD14 expression was significantly diminished in patients with SARS-CoV-2 infection compared to patients without the infection in the Mann–Whitney U test (difference ± SD; 405.90 ± 177.40 MFI; *p* = 0.03), whereas the counts of the CD16 + monocytes were significantly higher (difference ± SD; 0.07 ± 0.03 per nl; *p* = 0.01) (Figure 3A,B). The ROC curves showed an AUC of 0.76 (95% CI 0.55–0.98, *p* = 0.03) for the CD14 expression and an AUC of 0.81 (95% CI 0.64–0.98, *p* = 0.01) (Figure 3C,D) for the CD16 + monocyte count. Together, our findings indicate that the CD16 + monocyte count is the most robust diagnostic parameter for identifying a SARS-CoV-2 infection in the myeloid cell compartment.

### 3.3. Immunological Characteristics of Severe COVID-19 Infection

**Figure 4:**
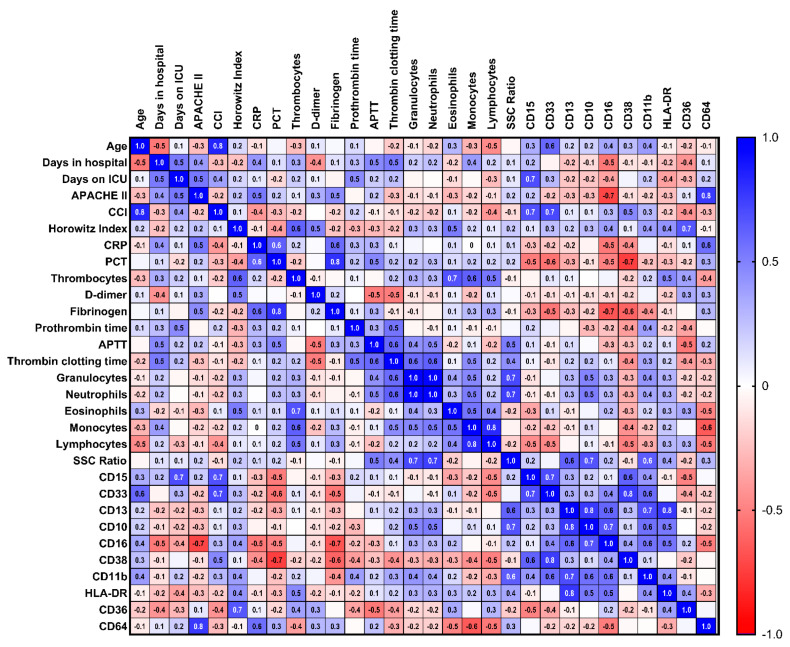
**Correlation matrix of the neutrophil granulocytes and clinical parameters.** Correlation matrix (heat map) of the immunophenotypic characteristics of the neutrophil granulocytes and clinical parameters of patients with COVID-19 in the ICU (*n* = 12). Spearman’s ρ ranks between ± 1.0 (dark blue, dark red), high (inverse) correlation, and ± 0 (white), no correlation (abbreviations: ICU = intensive care unit; *n* = number; APACHE = acute physiology and chronic health evaluation; CCI = Charlson comorbidity index; CRP = C-reactive protein; PCT = procalcitonin; APTT = activated partial thromboplastin time; SSC = side-scatter).

**Figure 5:**
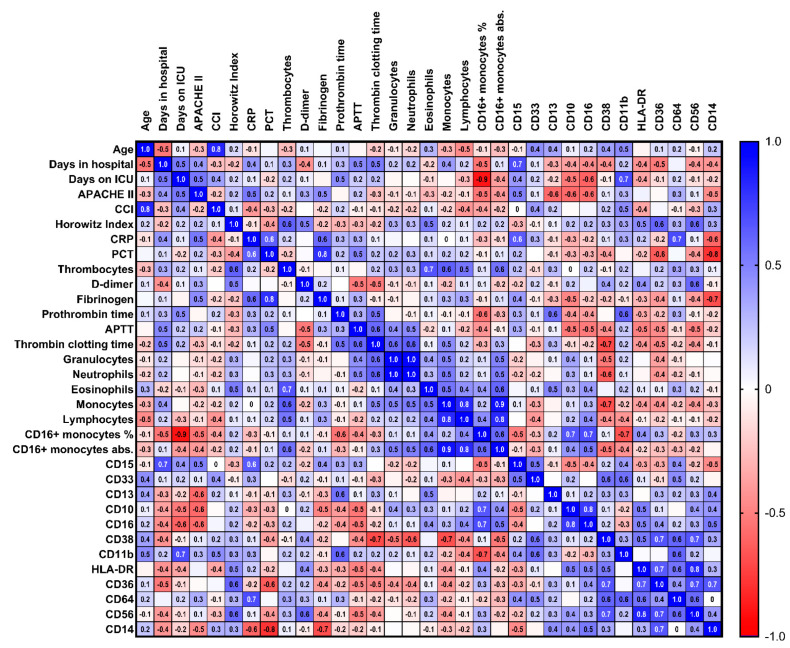
**Correlation matrix of the monocytes and clinical parameters.** Correlation matrix (heat map) of the immunophenotypic characteristics of the monocytes and clinical parameters of patient with COVID-19 in the ICU (*n* = 12). Spearman’s ρ ranks between ± 1.0 (dark blue, dark red), high (inverse) correlation, and ± 0 (white), no correlation (abbreviations: ICU = intensive care unit; *n* = number; APACHE = acute physiology and chronic health evaluation; CCI = Charlson comorbidity index; CRP = C-reactive protein; PCT = procalcitonin; APTT = activated partial thromboplastin time; SSC = side-scatter).

Next, we turn to the immunological differences between the patients with or without SARS-CoV-2 infection. We searched for markers of a severe course of COVID-19 infection within the patients who were admitted to an ICU (*n* = 12). The disease severity was assessed based on the APACHE-II score and Horowitz- index, which are related to the morbidity and mortality of ICU patients and the degree of ARDS, respectively. In addition, immunological characteristics were correlated with other clinical parameters, such as age, number of days in the hospital or ICU, the Charlson comorbidity index, inflammation (C-reactive protein and procalcitonin), coagulation (d-dimer, thrombocytes, fibrinogen, prothrombin time, activated partial thromboplastin time, and thrombin clotting time). The Spearmen’s correlation matrix results for the immunophenotypic characteristics of the neutrophil granulocytes are depicted in Figure 4, and those for the monocytes are depicted in Figure 5. Severe COVID-19 infection was correlated with a low CD16 expression (r = (−) 0.72; 95% CI (−)0.92–(−)0.23; *p* = 0.01) and high CD64 expression (r = 0.76; 95% CI 0.31–0.93; *p* = 0.01) in the neutrophil granulocytes, and ARDS was correlated with CD36 expression (r = 0.66, 95% CI 0.07–0.91; *p* = 0.03).

In the monocyte compartment, there was no significant correlation with the APACHE-II score or Horowitz index. However, the CD16 expression on the monocytes fell only slightly short of a significant correlation (r = (−) 0.58; 95% CI (−) 0.87–0.02; *p* = 0.05) and CD64 clearly had a correlation (*p* = 0.32). The CD36 expression on the monocytes fell slightly short of a significant correlation with the Horowitz index (r = 0.60; 95% CI (−) 0.03–0.89; *p* = 0.06).

Finally, we ruled out the possibility that the immunophenotypic findings are not generally associated with a severe course of disease in the ICU (“ICU phenomena”). Therefore, we excluded a correlation between the expression of CD16 and CD64 with the APACHE-II score (*p* = 0.10 and *p* = 0.30) and the Horowitz index with CD36 (*p* = 0.71) in ICU patients without SARS-CoV-2 (data not shown).

## 4. Discussion

In this study, we showed that the circulating neutrophil granulocytes of patients with COVID19 pneumonia have significantly a lower granularity and expression of CD15, whereas monocytes express decreased levels of CD14. Furthermore, the counts of the monocyte subsets with CD16 expression, which belong to the non-classical and intermediate monocyte categories, were significantly increased in patients with COVID-19. Our results are in line with previous reports that described a dysregulated myeloid cell compartment in cases of severe COVID-19 disease [28,32]. More specifically, Zhou et al. and Silvin et al. identified a higher proportion of the intermediate monocyte subset in COVID-19 disease than in the controls [32,33]. Interestingly, the SARS-CoV-2 S1 protein persists in CD16-positive monocytes after infection and might be associated with post-acute sequelae SARS-CoV-2 infection (long COVID) [34].

The lower expression of CD15 on neutrophils has been reported in patients with COVID-19 [35,36] and was confirmed by our results. CD15 is important for the transendothelial migration of the neutrophils [37,38], and the downregulation of CD15 might be associated with endothelial dysfunction in COVID-19 [39]. Reduced granularity of the neutrophil granulocytes has been described in terms of COVID-19 [40,41], but it is also common in other inflammatory states [42]. Our results gain are bolstered by the consistently selected control group of patients with pulmonary infiltrates but without SARS-CoV-2 infection, who were admitted to hospital within the same period of time. Therefore, our findings shed additional light on the specific immunophenotypes of the myeloid cells in COVID-19 compared to other forms of pneumonia, rather than on a more general difference between a pathologic state and the healthy state of controls.

A low CD16 expression and high CD64 expression on the neutrophil granulocytes was correlated with the severity of the health condition and risk of death (a high APACHE-II score) in patients with COVID-19 in the ICU. This finding is, at first glance, contradictory, because both antigens are Fc-gamma receptors, which bind the IgG molecules within an immune complex. Yet, CD16 is downregulated through proteolysis upon activation and apoptosis [43,44,45], and CD64 is upregulated through interferon γ (IFNγ) and the granulocyte colony-stimulating factor (G-CSF) in the neutrophil granulocytes [46,47]. CD16 is classified as a low-affinity Fc-gamma receptor III (FcgRIII), and CD16b/FcgRIIIb is solely expressed on the neutrophil granulocytes [48]. In the case of FcgRIIIb ligation to the unspecific Fc region of the antibodies (e.g., IgG) or IgG aggregates, intracellular granules from the neutrophils [49,50] are released. Ankerhold et al. described that soluble circulating IgG immune complexes in SARS-CoV-2 infection, which might contribute to an enhanced CD16b/FcgRIIIb activation that results in a “vicious cycle of escalating immunopathology“ [51]. This might explain the lower expression of CD16b/FcgRIIIb in COVID-19 patients, because the activation of CD16b/FcgRIIIb results in the downregulation of CD16b/FcgRIIIb [43,44,45].

CD64 is classified as a high-affinity Fc-gamma receptor I (FcγRI) and is normally expressed on the monocytes. It is an indicator of sepsis and might be associated with higher mortality in the ICU [48,48,52,53,54,55]. We propose that a low CD16 expression on the neutrophil granulocytes might indicate a severe COVID-19 infection, whereas a high CD64 expression more generally indicates a severe health condition because of its reactivity to IFNγ and G-CSF.

There are limitations to this study. Only patients of the first COVID-19 wave between March and May 2020 in Germany were included. SARS-CoV-2 variants, vaccination statuses, and COVID-19 treatment have changed since then, and these factors might have an impact on the immunopathology of COVID-19 disease. Therefore, our results have to be confirmed in study based on a larger patient cohort and with the new variants of SARS-CoV-2. The disease severity was quantified using the APACHE II score in this study. APACHE II correlates with the hospital death of ICU patients in general [29]. Other disease severity scores might be more specific to patients with COVID-19 pneumonia [56,57,58].

## 5. Conclusions

In this study, we showed that neutrophil granulocytes with a low CD15 expression and diminished granularity were characteristic of COVID-19 pneumonia. The severity of COVID-19 infection was correlated with a low expression of CD16 (FcgRIIIb) and high expression of CD64 (FcγRI) on the neutrophil granulocytes. This indicates the important role of Fc-gamma receptors in severe COVID-19 pneumonia.

## Figures and Tables

**Table 1:** Patient characteristics.

Characteristic	COVID-19 Positive (*n* = 16)	COVID-19 Negative (*n* = 9)
Median age (range)—years	66.5 (23–82)	70 (28–88)
Male sex—no. (%)	13 (81%)	5 (56%)
Female sex—no. (%)	3 (19%)	4 (44%)
**Underlying Health Condition** **—No./Total No. (%)**		
Diabetes	6 (38%)	1 (11%)
Hypertension	11 (69%)	5 (56%)
Obesity (BMI ≥ 30)	4 (25%)	2 (22%)
Hyperlipidemia	3 (19%)	0 (0%)
Chronic heart disease	5 (31%)	1 (11%)
Cardiovascular disease	3 (19%)	4 (44%)
Respiratory disease	1 (6%)	3 (33%)
Chronic kidney disease	1 (6%)	3 (33%)
Cancer	3 (19%	3 (33%)
Immunosuppression	3 (19%)	1 (11%)
Chronic liver disease	2 (13%)	1 (11%)
Charlson Comorbidity Index -/median (range)	4 (1–7)	5 (0–11)
**Complications—No./Total No. (%)**		
Acute respiratory distress syndrome	11 (69%)	4 (44%)
Non-invasive ventilation	0 (0%)	2 (22%)
Invasive mechanical ventilation	11 (69%)	4 (44%)
Acute kidney failure	8 (50%)	2 (22%)
Thromboembolic events	3 (19%)	0 (0%)
ICU	12 (75%)	6 (67%)
Death	3 (19%)	1 (11%)

## Data Availability

Not applicable.
